# The Impact of Red Songs and Music Training Experience on Implicit Prosocial Attitudes: Evidence from the SC-IAT Paradigm and Event-Related Potentials

**DOI:** 10.3390/bs16040505

**Published:** 2026-03-28

**Authors:** Yongcan He, Bo Yang, Yong Liu, Shuo Wang, Maoping Zheng

**Affiliations:** 1 School of Psychology, Southwest University, Chongqing 400715, China; heyongcan@email.swu.edu.cn (Y.H.); yangbo1966@email.swu.edu.cn (B.Y.); liuy0768@swu.edu.cn (Y.L.); ws241674@email.swu.edu.cn (S.W.); 2 Faculty of Music and Dance, Zunyi Normal University, Zunyi 563000, China; 3 School of Music, Southwest University, Chongqing 400715, China; 4 School of Music, Guizhou Normal University, Guiyang 550025, China

**Keywords:** red songs, music training, implicit prosocial attitudes, SC-IAT, event-related potentials (ERPs), neurocognitive mechanisms

## Abstract

Prosocial behavior is a core element of social harmony, and implicit prosocial attitudes, which may outperform explicit assessments in predicting real-world behavior, underscore their unique utility in prosocial and moral research contexts. Moreover, red songs, a distinctive musical form emerging in specific revolutionary and developmental periods of China, align with this prosocial potential, as they are characterized by lyrics advocating patriotism, collective memory, and emotional resonance. However, the specific effect of red songs on implicit prosocial attitudes, as well as the potential moderating role of music training experience in this relationship, remains underexplored. This study aimed to explore whether red songs enhance implicit prosocial attitudes compared to neutral songs, whether music training modulates this effect, and the underlying neural correlates using the Single-Category Implicit Association Test (SC-IAT) and event-related potentials (ERPs). A mixed-factorial design was used with 60 college students (30 with ≥5 years of music training, 30 without). Participants completed the SC-IAT (measuring implicit prosocial D-scores) while EEG data were recorded, while listening to red (“China in the Lantern Light”) and neutral (“Lake Baikal”) songs. ERP components (N1, P2, N3, LPCs) were analyzed. Behaviorally, no significant main effects of song type or music training were observed, but a significant interaction emerged (F(1, 58) = 4.09, *p* = 0.04): the music training group showed higher D-scores under red songs (M = 0.35, SD = 0.32) than neutral songs (M = 0.15, SD = 0.51), while the non-music training group exhibited the opposite non-significant trend. Neurally, repeated measures ANOVAs revealed a significant main effect of electrode site for N1 (F(4, 212) = 48.63, *p* < 0.001, partial η^2^ = 0.48), with the largest amplitudes at FCz. Red songs elicited larger N1 amplitudes than neutral songs at Fz and FCz, and incongruent trials elicited larger N1 amplitudes at Pz. For P2, a main effect of condition was found (F(1, 52) = 7.02, *p* = 0.01), with larger amplitudes in incongruent trials, and a significant three-way interaction of song type, condition, and electrode site (F(4, 208) = 4.46, *p* = 0.006), with larger P2 amplitudes for red songs under incongruent trials at Fz. For N3, main effects of song type (F(1, 53) = 14.48, *p* < 0.001) and stimulus type (F(2, 106) = 8.32, *p* = 0.001) were observed; congruent trials elicited larger N3 amplitudes than incongruent trials at Fz and FCz. For LPCs, main effects of song type (F(1, 53) = 4.89, *p* = 0.03) and electrode site (F(4, 212) = 3.05, *p* = 0.047) were found, with the largest amplitudes at Pz and the smallest at FCz. Red songs enhance implicit prosocial attitudes specifically among individuals with music training, and are accompanied by multi-stage neurocognitive differences. These findings highlight the conditional effects of red songs and inform prosocial education.

## 1. Introduction

Prosocial behavior, defined as voluntary actions benefiting others or groups (Eisenberg et al., 1995; Schroeder & Graziano, 2015), is fundamental to social harmony. According to the dual-attitudes model (Wilson et al., 2000), evaluations can operate at explicit and implicit levels, which may differentially relate to behavior. When addressing sensitive constructs such as moral goodness, implicit measures offer a potentially more robust approach to mitigating participants’ response biases and capturing cognitive attitudes that are often inaccessible via explicit self-report (Hahn & Gawronski, 2015). Consistent with this, a growing body of empirical evidence discovered that implicit measures may outperform explicit assessments in predicting real-world behavior, underscoring their unique utility in prosocial and moral research contexts (Greitemeyer, 2009; Hofmann et al., 2005; Huntjens et al., 2014; Nosek et al., 2011). Studies also have found that using belief-related words for implicit priming to examine conceptual structures derived from memory can yield fewer biases when investigating socially desirable topics (e.g., prosocial behavior) (Pichon et al., 2007; Chen & Chen, 2022). “Red songs,” a culturally specific genre embodying revolutionary spirit and collective values (Zheng, 2021), ranging from classic works such as “The March of the Volunteers” (《义勇军进行曲》) during the revolutionary wars to contemporary compositions such as “China in the Lantern Light” (《灯火里的中国》), have been empirically linked to prosocial tendencies (He et al., 2025). And red songs are potentially considered an eliciting agent for self-transcendent emotions like awe (Keltner & Haidt, 2003; Konečni, 2008; Stellar et al., 2017; Yaden et al., 2019), a self-transcendent emotion that enhances helping and moral behaviors (Piff et al., 2015; Stellar et al., 2017), thereby promoting prosociality. This study explores whether red music, as a culturally specific form embodying revolutionary spirit and collective values, can serve as an effective pathway to promoting prosocial behavior by associating with implicit prosocial attitudes.

Music has long been recognized as a cultural medium that shapes prosocial cognition and behavior, with studies showing that exposure to songs with prosocial lyrics enhances both implicit and explicit prosocial behavior (Ding et al., 2015; Ruth, 2019). Red songs, a distinctive musical form emerging in specific revolutionary and developmental periods of China (Cheng, 2025; He et al., 2025; Ma & Sun, 2025; Qin, 2024), align with this prosocial potential. Characterized by lyrics advocating patriotism, collective memory, and emotional resonance, they vividly depict the arduous journey of the Chinese people, embodying revolutionary spirit, national ethos, and cultural values that emphasize collective welfare, social unity, and self-sacrifice for the greater good. As an irreplaceable cultural symbol carrying shared historical memories and collective identity, red songs hold distinct cultural and emotional significance in the Chinese context, with the potential to arouse strong national identity and emotional resonance among Chinese audiences (Qin, 2024; Cheng, 2025). This cultural specificity—rooted in China’s unique revolutionary history and national spirit—could potentially contribute to modulating social cognition (e.g., ideological identification, value shaping), though such effects, if present, may differ from those associated with other forms of prosocial music and remain to be empirically verified (Qin, 2024).

From another aspect, red songs, rooted in China’s revolutionary history and carrying communist beliefs (Zheng, 2021), embody core values like collective cooperation and dedication, and their emotional resonance can evoke ideological identification with such beliefs; meanwhile, Chen et al. reported through empirical studies that communist beliefs can significantly promote moral cognition and prosocial tendencies via implicit perceptual processes (Chen & Chen, 2022), which aligns with Zheng’s (2021) finding that red songs’ “moral and social emotions” guide individuals toward collective-oriented behaviors. Thus, red songs may indirectly enhance prosocial behavior by implicitly activating communist beliefs and further fostering moral cognitive tendencies. Moreover, recent studies have begun exploring the educational value of red music culture in fostering prosocial values among adolescents (Ma & Sun, 2025; M. Xu et al., 2025). In summary, the empirical evidence on the impact of red music on implicit prosocial attitudes is still limited.

Event-related potentials (ERPs), a neuroscientific technique with high temporal resolution, offer a powerful tool to dissect the time-course dynamics of information processing during prosocial decision-making (Carlson et al., 2016; Xiao et al., 2015). However, few studies have explored changes in implicit prosocial cognition and their underlying neurocognitive mechanisms following short-term music exposure—an important gap in the current literature. Notably, ERP components have demonstrated high sensitivity to distinct cognitive processes in the Implicit Association Test (IAT) paradigms. For instance, during the early stages of information processing, the N1 component (linked to selective attention and perceptual analysis) is sensitive to the congruence between stimuli and self-relevant judgments, with larger amplitudes elicited by stimuli that conflict with self-schemas (W. F. Li et al., 2015). For instance, at the mid-stage of prosocial stimulus processing, the P2 component, which peaks at 200 ms in the frontal region of the brain, is closely linked to implicit cognition. The brain automatically matches incoming visual information with internal representations, and P200 amplitude is positively correlated with the degree of this matching (Evans & Federmeier, 2007). At the same time, the P2 effect is usually related to the automatic processing of emotions without being affected by the depth of vocabulary processing, including passive viewing (Begleiter & Platz, 1969), subconscious presentation (Bernat et al., 2001), lexical decision-making tasks (Kanske & Kotz, 2007; Schacht & Sommer, 2009), or emotional assessment tasks.

Moreover, N3 is an EEG component used to study the emotional processing of visual stimuli (Carretié et al., 1997). In the latency range examined here, a centro-parietal negativity is discussed under different labels in the literature, including N2, N300, or N400, depending on task demands and scalp distribution (Hamm et al., 2002; Draschkow et al., 2018; Truman & Mudrik, 2018). In the present SC-IAT context, we refer to this component as N3 and define it operationally as a centro-parietal negativity in the 300–380 ms window. Rather than indexing early sensory encoding, prior work links negativities in this window to context-dependent perceptual–semantic integration, early semantic matching under incongruity, and conflict-related processing (Draschkow et al., 2018; McPherson & Holcomb, 1999; Mudrik et al., 2010; Hamm et al., 2002; Võ & Wolfe, 2013). Based on this framework, we examined whether red songs modulate this intermediate perceptual–semantic integration stage during the processing of prosocial-related stimuli in the SC-IAT. The late positive component (LPC) is a parietally distributed waveform emerging around 300 ms post-stimulus and persisting throughout stimulus presentation (Lou et al., 2021)—is another key index: prior research using IAT or IAT-like tasks has consistently shown that congruent trials (where target and attribute associations align) evoke significantly larger LPC amplitudes than incongruent trials, reflecting enhanced motivational and emotional processing of congruent information (Coates & Campbell, 2010; Forbes et al., 2012; Williams & Themanson, 2011). We argue that investigating the relationship between red music and implicit prosocial cognition, while providing neurocorrelational evidence (focusing on the N1, P2, N3, and LPCs in ERPs), may offer a valuable supplement to the existing literature and shed light on an understudied direction in the current field. This study will primarily center on the N1, P2, N3, and LPCs in event-related potentials (ERPs) to explore this potential association.

The influence of red songs on implicit prosocial attitudes may be moderated by individual differences in music training experience. Long-term music training has been shown to enhance emotional recognition abilities (Herholz & Zatorre, 2012), and group music training facilitates the development of prosocial skills. (Schellenberg et al., 2015). Individuals with music training show heightened sensitivity to emotional and semantic cues in music (Castro & Lima, 2014), which may amplify the implicit prosocial effects of red songs by facilitating deeper processing of both lyrical meaning and emotional expression. Additionally, empathy ability is an important factor in predicting and promoting prosocial behavior, and musical training plays an important role in the development of empathy ability. However, the moderating role of music training in the relationship between red songs and implicit prosocial behavior remains unclear, and its underlying neural mechanisms have not been examined.

Despite these theoretical foundations, several gaps exist in the current literature. First, although laboratory studies have shown short-term enhancements in implicit prosociality following music exposure (Q. Xu et al., 2025; Hong et al., 2023), the specific effects of red songs as distinct from generic music remain deeply unexplored. Second, the interactive effect of music training and red song type on implicit prosocial attitudes has received little attention, leaving open questions about whether musical expertise modulates the processing of red songs’ prosocial cues. Third, there is more research on behavioral measurement, and the neurocognitive basis of red song effects on implicit prosocial processing remains poorly understood. The Single-Category Implicit Association Test (SC-IAT; Karpinski & Steinman, 2006) is a reliable tool for measuring the strength of implicit associations between a single target category and an attribute dimension. Unlike the traditional IAT, which requires contrast categories, the SC-IAT overcomes this limitation, making it particularly suitable for assessing implicit attitudes toward targets where clear contrast categories are unavailable or inappropriate. Combined with event-related potentials (ERPs), which offer high temporal resolution, the SC-IAT opens up the possibility of revealing the time-course neural dynamics of implicit prosocial processing (Q. Xu et al., 2025).

This study employs the SC-IAT paradigm and ERP technology to address the following questions: (1) whether red songs can enhance implicit prosocial attitudes compared to neutral songs; (2) whether music training experience modulates the implicit prosocial effects of red songs; and (3) what the neural correlates of these effects are, as reflected by ERP components (e.g., N1, P2, N3, LPC). Additionally, the study examines the correlation between implicit and explicit prosocial tendencies to examine the strength of the association. The findings are expected to advance our understanding of the culturally specific effects of red songs and the moderating role of musical expertise, while also possibly clarifying the implications of red music application in prosocial intervention and education.

Based on the theoretical and empirical evidence reviewed above, the present study proposes the following hypotheses:

**Hypothesis** **1.** *Exposure to red songs will enhance implicit prosocial attitudes compared to neutral songs, as reflected by higher SC-IAT D-scores.*

**Hypothesis** **2.** *Music training experience will moderate this effect, such that the prosocial enhancement induced by red songs will be stronger among individuals with formal music training than among those without.*

**Hypothesis** **3.** *Red songs will modulate neural processing of implicit prosocial information, as reflected in ERP components. Specifically, red songs are expected to enhance early attentional processing (N1), mid-stage attentional and emotional matching processes (P2, N3), and late motivational–emotional encoding (LPC).*

**Hypothesis** **4.** *Implicit prosocial attitudes (SC-IAT D-scores) will not be strongly correlated with explicit prosocial tendency scores, consistent with the dual-attitude framework.*

## 2. Materials and Methods

### 2.1. Participants

A priori power analysis was conducted using G*Power 3.1 (Faul et al., 2007) to determine the sample size: for a mixed ANOVA with medium effect size (f = 0.25), statistical power of 80%, and α = 0.05, at least 56 participants were required. A total of 72 college students were initially recruited, with 12 excluded (5 due to SC-IAT error rate > 20%, 7 due to incorrect judgment of experimental conditions). The final valid sample consisted of 60 participants (31 males, 29 females). The music training group (*n* = 30) had received at least 5 years of continuous formal music training, which included either instrumental or vocal practice such as piano, violin, or chorus participation. The classification was based on duration of formal instruction rather than specific training modality. In contrast, the non-music training group (*n* = 30) had no formal music training experience, with their only exposure to music being basic music education provided in primary and secondary school. All participants had normal or corrected-to-normal vision, normal hearing, no history of neurological disorders, and were right-handed; they provided written informed consent before the experiment and received ¥50 monetary compensation after completion. The study was approved by the Institutional Ethics Committee of Southwest University (approval number: H24153).

### 2.2. Experimental Design

The study employed a mixed-factorial design using the SC-IAT. Participants completed SC-IAT tasks under two song conditions (red songs and neutral songs). The task included two response conditions (congruent and incongruent) and three stimulus categories (prosocial words, non-prosocial words, and self-related concept words). Music training experience was treated as a between-subjects factor, with participants assigned to either the music training group or the non-music training group. Behavioral and neurophysiological data were collected to examine the effects of song type and music training experience on implicit prosocial attitudes.

### 2.3. Procedure

The experiment was programmed in E-Prime 2.0 for stimulus presentation and data collection. Participants completed the experiment individually in a sound-attenuated, temperature-controlled laboratory. After EEG cap preparation, participants first completed the Prosocial Tendencies Measure questionnaire. The experiment consisted of two sessions separated by a two-week interval to minimize potential carry-over effects. In each session, participants were exposed to one type of music (a red song or a neutral song). The order of music conditions and the SC-IAT block order were randomized and counterbalanced across participants. In each session, a 3 min music excerpt was presented while participants listened passively. The SC-IAT task began 300 ms after the offset of the music. Each SC-IAT consisted of two blocks (a congruent task and an incongruent task), and the order of these blocks was counterbalanced across participants. Each block contained 24 practice trials followed by 72 test trials. In a typical trial, a fixation cross appeared for 500 ms at the center of the screen. A word was then presented until participants responded, followed by a blank screen for 500 ms. In the congruent task, participants were asked to press the “F” key with their left index finger when they saw a self-related concept word or a prosocial word, and press the “J” key with their right index finger when they saw a non-prosocial word. In the incongruent task, participants were asked to press the left-hand response “F” key if the stimulus was a prosocial word, and press the right-hand response “J” key if the stimulus was a self-related concept word or a non-prosocial word. In order to prevent response bias, self-related concept words, prosocial words, and non-prosocial words were presented in a 7:7:10 ratio in the congruent condition, and there was a 7:10:7 ratio (self-related concept words, prosocial words, and non-prosocial words) in the incongruent condition, such that the corresponding correct responses were approximately 42% for the “F” key and 58% for the “J” key. For each trial, the stimulus remained on the screen until the participant responded. The maximum presentation duration is 1500 ms. If the presentation time exceeds this limit, a “Please respond as soon as possible” prompt will be displayed for 500 ms. Finally, there was feedback for 150 ms, in which a green “√” was presented when the participants’ response was correct; otherwise, a red “×” was presented (Wen & Zuo, 2007). After completing both sessions and the post-test Prosocial Tendencies Measure questionnaire, participants were debriefed, and the EEG cap was removed.

### 2.4. Materials

Experimental materials included two categories: lexical materials and music materials, with their screening and evaluation processes designed to ensure validity, reliability, and standardization, as detailed below.

#### 2.4.1. Stimuli Selection

Lexical materials were composed of concept words and attribute words. Concept words were self-related two-character Chinese words, while attribute words covered two semantic categories: prosocial and non-prosocial. The screening process strictly adhered to the principles of semantic consistency and standardized evaluation: First, preliminary screening was conducted based on “A Frequency Dictionary of Common Modern Chinese Words.” A total of 60 prosocial words and 60 non-prosocial words were initially selected, along with 120 neutral words as a control corpus, ensuring consistency in word frequency and part of speech (all two-character words) across categories to avoid confounding effects from these variables. Subsequently, 8 postgraduate students trained in systematic psychological research methods were invited to conduct meaning judgment and inter-rater reliability assessment of all selected words. The inter-rater reliability was evaluated using the Kappa coefficient, with a threshold of Kappa ≥ 0.85 to ensure high consistency in classification. Words with ambiguous semantics or significant disputes in classification were excluded, ultimately forming a candidate lexical set consisting of 40 prosocial words, 40 non-prosocial words, and 40 neutral words. To further verify the validity of the candidate words, 30 adult participants unrelated to the study (12 males, 18 females, aged 18~30 years, M = 23.56 years, SD = 2.18) were recruited to rate the words on three dimensions using a 5-point Likert scale (1 = lowest, 5 = highest): relevance (the degree of fit between the word and its corresponding semantic category), familiarity (frequency of daily exposure), and arousal (intensity of emotional activation triggered by the word).

Statistical analysis of the ratings showed that all words achieved a relevance score of ≥3 points, indicating clear semantic attribution and meeting the requirement of explicit category membership. Finally, based on the ranking of comprehensive scores (combining relevance, familiarity, and arousal), the top 10 prosocial words and top 10 non-prosocial words were selected, along with 6 self-related concept words, to form the final experimental lexical library.

Here are the final lexical list and its evaluation results. Prosocial words are as follows:爱心 (compassion), 尊重 (respect), 帮忙 (help), 关怀 (care), 团结 (unity), 分享 (share), 友善 (kindness), 鼓励 (encouragement), 帮助 (assistance), and 关心 (concern); non-prosocial words are as follows: 加害 (harm), 辱骂 (insult), 拐骗 (abduct), 攻击 (attack), 冷漠 (indifference), 欺辱 (humiliate), 欺凌 (bully), 逼迫 (coerce), 霸占 (seize), and 虚伪 (hypocrisy); and self-related concept words are as follows: 自己 (oneself), 自我 (self), 咱们 (we), 我们 (us), 我们的 (our), and 本人的 (myself). For prosocial words, their attribute scores were as follows: for relevance, M = 4.18 (SD = 0.58); for familiarity, M = 4.30 (SD = 0.58); and for arousal, M = 3.70 (SD = 0.61). For non-prosocial words, the corresponding attribute scores were as follows: for relevance, M = 4.11 (SD = 0.52); for familiarity, M = 4.23 (SD = 0.44); and for arousal, M = 3.71 (SD = 0.60).

#### 2.4.2. Music Materials

Music materials were divided into two types: red songs and neutral songs. The core screening principle was opposite thematic attributes and matched irrelevant variables, ensuring that only thematic content and emotional valence differed between the two types, while other objective characteristics were consistent to control for confounding factors.

Red songs were selected from the “100 Excellent Red songs” library compiled by the Publicity Department of the CPC Central Committee in 2019, which focuses on eulogizing the Party, the motherland, the people, and heroes, and combines ideological and artistic value. The specific screening criteria included: (1) distinct theme (highlighting positive social values such as patriotism and collectivism); (2) positive and high-spirited emotional expression; and (3) high public familiarity (referring to playback volume and popularity data on major music platforms). Finally, “China in the Lantern Light” was determined as the red song stimulus, with a 3 min core melody fragment extracted (consistent with the conventional duration standard for red song dissemination in psychological experiments).

Neutral songs were selected based on the standardized emotional music library (integrating domestic emotional music databases and the DEAM dataset) and popular music libraries of major domestic music platforms (NetEase Cloud Music, QQ Music). The screening logic formed a strict control with red songs, and the specific criteria were as follows. Thematic dimension: focusing on daily emotions, natural landscapes, and other non-social significance scenarios, without explicit value orientation or collectivist cues; emotional dimension: stable emotional expression (both pleasure and arousal at medium levels), without strong positive or negative emotional tendencies; matched variables: consistent with the red songs in duration (3 min fragment), rhythm (slow rhythm, BPM ≤ 80), familiarity (assessed by 30 participants in a pre-experiment, familiarity M = 4.21, SD = 0.63, with no significant difference from the red songs, t = 1.32, *p* > 0.05), and audio quality (lossless format, sampling rate 44.1 kHz); and final determination: considering melodic fluency, neutral emotional characteristics, and listenability, “Lake Baikal” was selected as the neutral songs stimulus. This piece is a major-key lyrical work, adopting neutral vibrato techniques with weak emotional orientation. Its theme focuses on natural landscapes and personal memories, without obvious social significance. Its emotional arousal (M = 3.32, SD = 0.75) was significantly lower than that of the red songs (M = 4.89, SD = 0.57), meeting the definition of a neutral stimulus.

### 2.5. Measures

#### 2.5.1. Behavioral Measures

Behavioral performance in the SC-IAT was assessed using reaction time, accuracy, and the D-scores, with the D-scores serving as the primary index of implicit prosocial attitudes. SC-IAT data were processed following the method used by Karpinski and Steinman (2006). Participants with an overall error rate greater than 20% were excluded. Then, trials with reaction times greater than 10,000 ms or less than 350 ms were removed. Incorrect responses were corrected by replacing their reaction time with the mean correct reaction time of the corresponding block plus a 400 ms penalty. The difference between the mean reaction times of congruent and incongruent trials was then divided by the standard deviation of all correct responses (excluding the original incorrect trials) to obtain the SC-IAT D-scores, which represent implicit prosocial attitudes.

#### 2.5.2. ERP Measures

ERP measures included the mean amplitudes of four components of interest: N1, P2, N3, and LPC.

#### 2.5.3. Questionnaire

Explicit prosocial tendencies were assessed using the Prosocial Tendencies Measure (PTM; Kou et al., 2007), a 26-item self-report scale covering six dimensions of prosocial behavior. Higher scores indicate stronger explicit prosocial tendencies. In the current study, the PTM demonstrated high internal consistency (Cronbach’s α = 0.95).

### 2.6. EEG Recording

EEG data were recorded using a NeuroScan SynAmps 2 system (Compumedics NeuroScan Inc., Abbotsford, VIC, Australia). A 64-channel Ag/AgCl electrode cap arranged according to the international extended 10–20 system was used to record the EEG signals. Vertical electrooculogram (VEOG) was recorded using electrodes placed above and below the right eye, and horizontal electrooculogram (HEOG) was recorded using electrodes positioned at the outer canthi of both eyes. The online band-pass filter was set at 0.05–400 Hz, and the sampling rate was 1000 Hz. All electrodes were referenced online to the left mastoid, with the ground electrode located between FPz and Fz. Electrode impedances were maintained below 5 kΩ.

### 2.7. EEG Data Processing

EEG preprocessing was conducted in MATLAB R2022a using the EEGLAB toolbox (Delorme & Makeig, 2004). A band-pass finite impulse response (FIR) filter ranging from 0.1 to 40 Hz was applied to the data. The data were re-referenced offline to the averaged left and right mastoids. EEG data were then segmented into trials spanning from −200 ms to 1000 ms time-locked to the onset of each word stimulus presented in the SC-IAT task. Data underwent baseline correction from −200 ms to 0 ms relative to this stimulus onset and were inspected on a trial-by-trial basis for quality assessment. Epochs containing excessive noise or abnormal fluctuations were rejected following visual inspection. Bad channels were interpolated using neighboring electrodes when necessary. Independent component analysis (ICA) was applied to identify and remove components reflecting ocular and other physiological artifacts. Subsequent to visual inspections of the ICA outcomes, components associated with EOG artifacts or head movements were excluded.

Of the 60 participants included in the behavioral analyses, six were excluded from the ERP analyses due to insufficient EEG data quality. Specifically, three participants were excluded because excessive artifacts resulted in more than 30% of trials being rejected after preprocessing, two participants were excluded due to electrode detachment during recording, and one participant was excluded due to data loss caused by technical failure. These exclusion criteria were established prior to statistical analyses and were applied uniformly across participants. The final ERP sample, therefore, consisted of 54 participants. Based on previous studies (Y. Liu et al., 2019; Zhou et al., 2022; Braboszcz & Delorme, 2011; Jing et al., 2024) and the grand-averaged ERP waveforms, mean amplitudes for N1 (100–170 ms), P2 (180–240 ms), N3 (300–380 ms), and LPCs (500–1000 ms) at electrodes Fz, FCz, Cz, CPz, and Pz were extracted for analysis.

### 2.8. Statistical Analysis

#### 2.8.1. Behavioral Analysis

Statistical analyses of the behavioral data were conducted using SPSS 25.0. Paired-samples *t*-tests were used to compare mean reaction times between congruent and incongruent conditions in the SC-IAT. One-sample *t*-tests were conducted to examine whether D-scores were significantly greater than 0 under each song condition. To examine the effects of song type and music training experience on implicit prosocial attitudes, a 2 (music training experience: music training group and non-music training group) × 2 (song type: red songs and neutral songs) mixed ANOVA was conducted on D-scores. Pearson’s correlation analyses were conducted to examine the association between explicit prosocial tendencies (PTM scores) and implicit prosocial attitudes (D-scores). Statistical significance was set at *p* < 0.05.

#### 2.8.2. ERP Analysis

For the ERP data, a 2 (music training experience: music training group and non-music training group; between-subjects) × 2 (song type: red songs and neutral songs; within-subjects) × 2 (condition: congruent and incongruent; within-subjects) × 3 (stimulus type: prosocial, non-prosocial, and self-related concept words; within-subjects) × 5 (electrode site: Fz, FCz, Cz, CPz, and Pz) repeated measures ANOVA was conducted for the mean amplitudes of N1, P2, N3, and LPCs. Greenhouse–Geisser corrections were applied when the assumption of sphericity was violated, and post hoc comparisons were Bonferroni-corrected. Statistical significance was set at *p* < 0.05.

## 3. Results

### 3.1. Implicit Prosocial Attitudes (SC-IAT Behavioral Results)

Paired-samples *t*-tests were conducted to compare the mean reaction times of participants under the consistent (i.e., concept words matched with attribute words) and inconsistent conditions, following their exposure to red songs and neutral songs, respectively. The results indicated a significant difference in reaction times between the congruent and incongruent tasks across these conditions:

After listening to red songs, the mean reaction time in the incongruent task was significantly longer than that in the congruent task (*t*(59) = 6.47, *p* < 0.001, Cohen’s *d* = 0.835); specifically, in the congruent task, participants’ mean reaction time was M = 707.71 (SD = 172.13), whereas it was M = 768.22 (SD = 191.09) in the incongruent task. The mean D-scores (computed using the specified formula) was M = 0.31 (SD = 0.35), and a one-sample *t*-test comparing D-scores to 0 revealed that D-scores were significantly greater than 0 (*t*(59) = 6.851, *p* < 0.001, Cohen’s *d* = 0.884), a finding that indicates participants responded faster when prosocial concept words were paired with self-related concept words, thereby confirming the existence of the implicit prosocial self-concept evaluation effect.

After listening to neutral songs, the mean reaction time in the incongruent task was also significantly longer than that in the congruent task (*t*(59) = 3.137, *p* = 0.003, Cohen’s *d* = 0.405): in this case, the mean reaction time in the congruent task was M = 765.79 (SD = 187.67), while that in the incongruent task was M = 822.62 (SD = 174.08); the mean D-scores (calculated via the same formula) were M = 0.24 (SD = 0.48), and a one-sample *t*-test demonstrated that D-scores were significantly greater than 0 (*t*(59) = 7.16, *p* < 0.001, Cohen’s *d* = 0.519), which similarly verifies that participants exhibited faster responses when prosocial concept words matched self-related concept words, i.e., the implicit prosocial self-concept evaluation effect was present.

Descriptive statistics for the four cells were as follows: in the music training group, D-scores were M = 0.35 (SD = 0.32) after red songs and M = 0.15 (SD = 0.51) after neutral songs; in the non-music training group, D-scores were M = 0.26 (SD = 0.36) after red songs and M = 0.34 (SD = 0.43) after neutral songs. These values are reported to describe the pattern of means, whereas inferential interpretation was based on the mixed ANOVA and follow-up simple-effects tests.

To test these patterns statistically, a 2 × 2 mixed-design ANOVA was conducted on D-scores, with music training experience as the between-subjects factor and song type as the within-subjects factor.

Mixed-design ANOVA results indicated that neither the main effect of music training experience (F(1, 58) = 0.41, *p* = 0.52, η_p_^2^ = 0.01) nor the main effect of song type (F(1, 58) = 0.67, *p* = 0.41, η_p_^2^ = 0.01) reached statistical significance, suggesting no overall differences in D-scores across training groups or song types.

**Figure 1:**
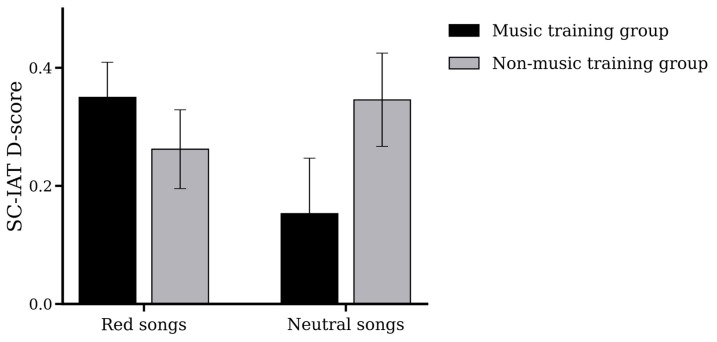
D-scores on the SC-IAT for participants with and without music training under red songs and neutral songs conditions (error bars represent standard errors). Note: black = music training group; gray = non-music training group.

Importantly, a significant interaction effect was observed between music training experience and song type (F(1, 58) = 4.09, *p* = 0.04, η_p_^2^ = 0.07). To further interpret this interaction, we compared the difference in D-scores between red and neutral conditions across groups. The red-minus-neutral difference score was significantly larger in the music training group (Mdiff = 0.20, SD = 0.54) than in the non-music training group (Mdiff = −0.08, SD = 0.53), *t*(58) = 2.02, *p* = 0.048, Cohen’s *d* = 0.52. Follow-up within-group paired comparisons showed that, in the music training group, D-scores were numerically higher after red songs than after neutral songs, but this difference did not reach significance, *t*(29) = 1.99, *p* = 0.056, Cohen’s dz = 0.36. In the non-music training group, the simple effect of song type was also not significant, *t*(29) = −0.86, *p* = 0.396, Cohen’s dz = 0.16. Thus, the interaction indicates that the direction and magnitude of the song-type effect differed by music training experience, rather than a uniform main effect of song type (see Table 1 and Figure 1).

### 3.2. ERP Results

**Figure 2:**
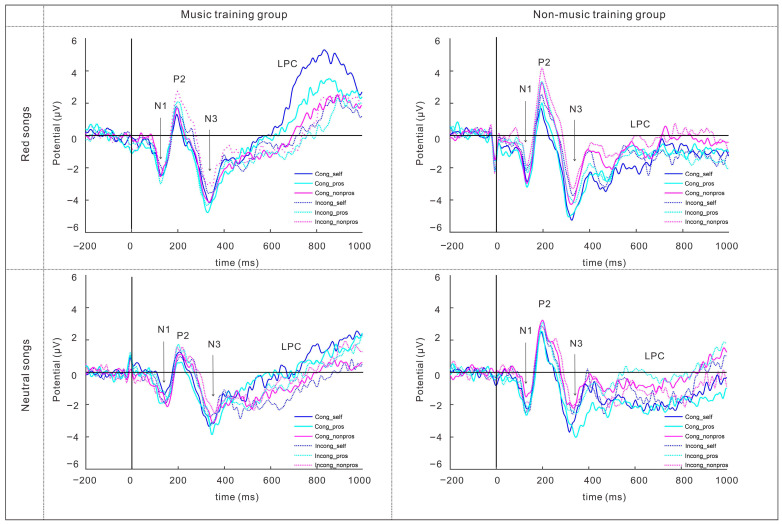
ERP waveforms (N1, P2, N3, LPCs) for SC-IAT congruent/incongruent trials: cross-comparisons of music/non-music training groups and red/control songs conditions.

Repeated measures ANOVAs were conducted to examine the effects of experimental factors (song type, stimulus type, condition, music training experience) on the amplitudes of four key ERP components (N1, P2, N3, LPCs) across five midline electrodes (Fz, FCz, Cz, CPz, Pz), with the corresponding waveform patterns visualized in Figure 2.

For the N1 component, a significant main effect of electrode site was observed, F(4, 212) = 48.63, *p* < 0.001, partial η^2^ = 0.48, indicating that N1 amplitudes varied across scalp locations, with the largest amplitudes at FCz and the smallest at Pz. In addition, the interaction between song type and electrode site was significant, F(4, 212) = 4.01, *p* = 0.03, partial η^2^ = 0.07. Simple-effects analyses showed that red songs elicited larger N1 amplitudes than neutral songs at Fz and FCz. The interaction between condition and electrode site was also significant, F(4, 212) = 6.72, *p* = 0.002, partial η^2^ = 0.11, with incongruent trials eliciting larger N1 amplitudes than congruent trials at Pz.

For the P2 component, the main effect of condition was significant, F(1, 52) = 7.02, *p* = 0.01, partial η^2^ = 0.12, with larger amplitudes in the incongruent condition than in the congruent condition. The main effect of stimulus type was also significant, F(2, 104) = 12.45, *p* < 0.001, partial η^2^ = 0.19. Post hoc comparisons showed that non-prosocial words elicited the largest P2 amplitudes, followed by prosocial words and then self-related concept words. Moreover, a significant three-way interaction of song type, condition, and electrode site was found, F(4, 208) = 4.46, *p* = 0.006, partial η^2^ = 0.08. Simple-effects analyses further indicated that, under the incongruent condition, red songs elicited larger P2 amplitudes than neutral songs at Fz.

For the N3 component, a significant main effect of song type was found, F(1, 53) = 14.48, *p* < 0.001, partial η^2^ = 0.22, with red songs eliciting larger N3 amplitudes than neutral songs. A significant main effect of stimulus type was also observed, F(2, 106) = 8.32, *p* = 0.001, partial η^2^ = 0.14, indicating that prosocial words elicited the largest N3 amplitudes, whereas self-related words elicited the smallest. In addition, the interaction between condition and electrode site was significant, F(4, 212) = 18.04, *p* < 0.001, partial η^2^ = 0.25. Simple-effects analyses showed that congruent trials elicited larger N3 amplitudes than incongruent trials at Fz and FCz.

**Figure 3:**
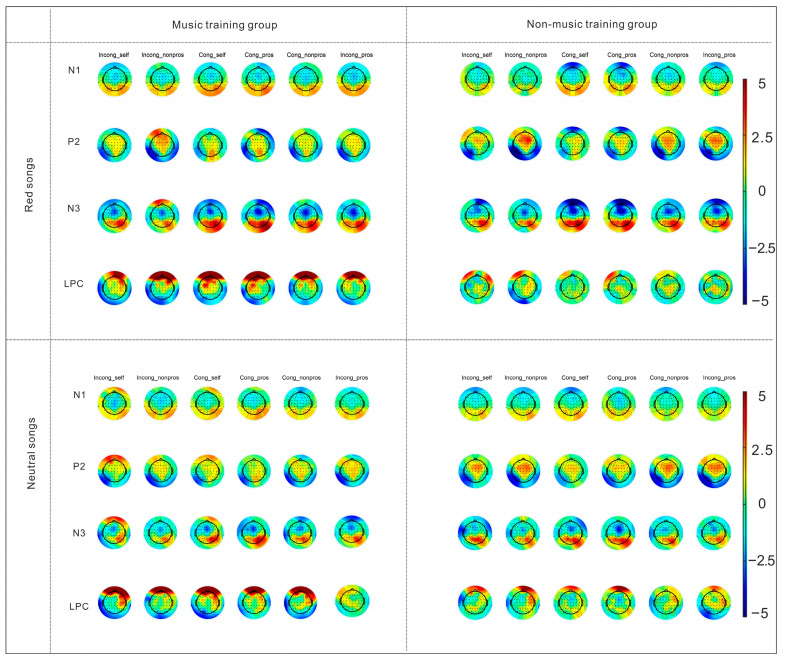
Topographic maps of ERP components (N1: 100–170 ms; P2: 180–240 ms; N3: 300–380 ms; LPCs: 500–1000 ms) for red and neutral song conditions in the music training and non-music training groups.

For the late positive component (LPC), a significant main effect of song type was observed, F(1, 53) = 4.89, *p* = 0.03, partial η^2^ = 0.08, with larger LPC amplitudes following red songs than neutral songs. A significant main effect of electrode site was also found, F(4, 212) = 3.05, *p* = 0.047, partial η^2^ = 0.05, indicating that LPC amplitudes differed across electrode sites. Post hoc comparisons showed that the largest LPC amplitudes were observed at Pz, whereas the smallest amplitudes were observed at FCz. Topographic maps for the time windows of N1 (100–170 ms), P2 (180–240 ms), N3 (300–380 ms), and LPCs (500–1000 ms) are presented in Figure 3, providing a visualization of the spatial distribution of the observed ERP effects.

### 3.3. Correlation Between Explicit and Implicit Prosocial Attitudes

Pearson’s correlation analysis showed that explicit prosocial tendency scores were notsignificantly correlated with implicit prosocial attitude D-scores in either song condition (all *p* > 0.05), suggesting that the explicit and implicit measures may capture relatively distinctaspects of prosociality (see Table 2).

## 4. Discussion

The present study aimed to investigate the effects of red songs on implicit prosocial attitudes, the potential moderating role of music training experience, and the underlying neural correlates using the SC-IAT paradigm and ERP technology. The results revealed three core findings that collectively advance our understanding of the interplay between cultural music, individual differences, and implicit social cognition: no significant main effects of song type or music training experience on implicit prosocial attitudes, but a significant interaction between the two; distinct neural correlates of red song processing—specifically, red songs elicited larger N1 and N3 amplitudes than neutral songs, the P2 component showed a song type × condition interaction, and the music training group exhibited larger LPC amplitudes; and a clear dissociation between explicit prosocial behaviors and implicit prosocial attitudes. These findings not only address the research gaps identified in the introduction but also provide new insights into the neurocognitive mechanisms through which red songs modulate implicit prosocial attitudes.

### 4.1. Behavioral Effects: SC-IAT Results of Implicit Prosocial Attitudes

The SC-IAT behavioral results confirmed the robust existence of the implicit prosocial self-concept evaluation effect across both song conditions. Paired-samples *t*-tests revealed that regardless of whether participants were exposed to red songs or neutral songs, their mean reaction times in incongruent tasks (prosocial concepts paired with non-self attributes or vice versa) were significantly longer than those in congruent tasks (prosocial concepts matched with self-relevant attributes). Correspondingly, one-sample *t*-tests demonstrated that the D-scores (quantifying the strength of implicit association between prosocial concepts and self) were significantly greater than 0 in both conditions. This finding aligns with Ding et al. (2015), who found that implicit prosocial attitudes, as measured by the traditional IAT, reflect an automatic psychological structure that can be stably activated across different musical contexts, and further supports Q. Xu et al. (2025)’s conclusion that the SC-IAT paradigm is a reliable tool for capturing implicit prosocial attitudes by effectively indexing the associative strength between self and prosocial concepts. Notably, the consistent presence of implicit prosocial attitudes across both red and neutral song conditions echoes the observation that such attitudes are deeply embedded in social cognitive processing, showing stability even when exposed to different types of musical stimuli (Ding et al., 2015; Q. Xu et al., 2025). At the same time, implicit prosocial attitudes are potentially more predictive of real-world spontaneous helping behaviors than explicit self-reports (Greitemeyer, 2009; Hofmann et al., 2005; Huntjens et al., 2014; Nosek et al., 2011), and the consistent presence of this effect in the current study further underscores the ecological relevance of investigating implicit prosociality.

Critically, mixed-design ANOVA results revealed no significant main effects of song type or music training experience on D-scores, but a significant interaction effect between the two. More specifically, the difference in D-scores between red and neutral conditions was larger in the music training group than in the non-music training group, *t*(58) = 2.02, *p* = 0.048, Cohen’s *d* = 0.52. Follow-up within-group comparisons further showed that the difference in D-scores between red and neutral conditions in the music training group did not reach conventional significance, *t*(29) = 1.99, *p* = 0.056, Cohen’s dz = 0.36, and that the corresponding contrast in the non-music training group was also not significant, *t*(29) = −0.86, *p* = 0.396, Cohen’s dz = 0.16. Therefore, the interaction is best interpreted as evidence that song type operated differently across groups, rather than as evidence for a reliable simple effect in only one group.

One plausible interpretation is that individuals with formal music training may be more sensitive to the emotional and semantic cues embedded in red songs. For individuals with ≥5 years of formal music training, long-term structured practice enhances sensitivity to emotional and semantic cues in music (Castro & Lima, 2014; Herholz & Zatorre, 2012), enabling them to deeply perceive and integrate the collective-oriented, prosocial themes (e.g., patriotism, unity, self-sacrifice) embedded in red songs (Ma & Sun, 2025; M. Xu et al., 2025). This deep processing may be associated with enhanced activation of prosocial cognitive schemas (Greitemeyer, 2009; Ruth, 2019), which could contribute to a stronger implicit association between prosocial concepts and self-identity. However, given the arousal differences between stimuli, this interpretation should be considered tentative. In contrast, non-music trained individuals may lack such a cognitive scaffold to fully engage with the cultural and emotional intensity of red songs, leading to reduced activation of prosocial schemas. For this group, neutral songs—with their milder emotional tone and absence of value-laden content—do not interfere with inherent implicit prosocial attitudes (Ding et al., 2015; Greitemeyer, 2009), resulting in relatively higher D-scores under the neutral songs condition.

Additionally, Pearson’s correlation analysis confirmed a lack of significant associations between explicit prosocial tendency scores (PTM scores) and implicit measures (SC-IAT D-scores, ERP amplitudes) in either song condition (all *p* > 0.05). This dissociation provides robust evidence for the discriminant of implicit and explicit prosocial constructs, consistent with the dual-attitudes model (Wilson et al., 2000; Aydinli et al., 2014) and highlighting that implicit prosocial attitudes, as measured by the SC-IAT, reflect distinct cognitive processes that are not captured by self-report measures.

### 4.2. Neural Correlates: ERP Results of Implicit Prosocial Processing

The ERP results uncovered distinct neural mechanisms underlying the observed behavioral effects, with each component reflecting a unique stage of cognitive–emotional processing. The N1 component is measured at frontocentral sites within the 100–170 ms time window. It is linked to early selective attention and perceptual analysis, and sensitive to the congruence between stimuli and self-relevant judgments—larger amplitudes are typically elicited by stimuli that conflict with self-schemas (W. F. Li et al., 2015). For this component, no significant main effect of song type was observed for N1 amplitudes. However, a significant interaction between song type and electrode site indicated that red songs elicited larger N1 amplitudes than neutral songs, specifically at frontocentral electrodes (Fz and FCz). This indicates that red songs, with their culturally salient themes and higher emotional arousal, capture greater early perceptual attention in a spatially selective manner, rather than uniformly across the scalp during the processing of self/prosocial vocabulary. However, given the higher arousal ratings observed in the material validation phase, this enhancement may partially reflect arousal-driven modulation rather than content-specific processing alone. This potentially amplifies the detection of congruence or conflict between prosocial concepts and self-relevant schemas at the initial perceptual stage (W. F. Li et al., 2015). Thus, while red songs appear to influence early attentional processes, the present findings cannot conclusively disentangle the relative contributions of emotional arousal and culturally embedded meaning at this stage. Notably, no main effect of music training on N1 amplitudes was found, suggesting that early perceptual sensitivity to song salience may operate relatively automatically and is not strongly dependent on musical expertise.

Moving to the P2 component (180–240 ms, frontal sites), associated with a variety of cognitive tasks, including stimulus classification, response inhibition, selective attention, and short-term memory tasks (Bary et al., 2003; Crowley & Colrain, 2004; Key et al., 2005). The P2 component is mainly caused by visual stimuli and represents the components of the individual’s higher-order perceptual processing of attentional stimuli (Kanske et al., 2011), and a larger P2 amplitude also indicates more attention to the stimulus (Finnigan et al., 2011). The present results revealed a main effect of stimulus type, where non-prosocial words elicited larger amplitudes, reflecting increased attentional vigilance to negative/non-prosocial stimuli, and a main effect of condition with larger amplitudes in incongruent trials, indexing cognitive conflict during stimulus matching. Importantly, these effects were further qualified by a significant three-way interaction among song type, condition, and electrode site. Follow-up analyses indicated that under the incongruent condition, red songs elicited larger P2 amplitudes than neutral songs at frontal electrodes (Fz), suggesting that the modulation of conflict-related attention by red songs is spatially specific. This pattern suggests that the red songs may amplify attentional engagement with conflictual prosocial–self associations (Rodríguez-Gómez et al., 2020), although this enhancement may also be influenced by differences in emotional arousal between stimuli, potentially facilitating the resolution of these conflicts and strengthening implicit prosocial attitudes. In addition, positive music has been reported to elicit larger P2 amplitudes than negative music (Proverbio et al., 2020). Thus, the larger P2 amplitudes observed in the red song condition may also partly reflect its more positive emotional characteristics. This pattern suggests that red songs may be associated with modulation of mid-stage cognitive conflict processing, though arousal-related factors cannot be fully ruled out. This stage may represent an intermediate processing phase between early perceptual attention and later semantic integration, which could indirectly relate to variations in prosocial–self associations. Notably, no main effect of music training on P2 was found, indicating that this attentional modulation is driven by song type rather than individual differences in musical skill.

The N3 component (300–380 ms, central–parietal sites) is an EEG marker used to investigate the emotional processing of visual stimuli (Carretié et al., 1997) and has also been discussed in relation to context-dependent perceptual–semantic integration and early semantic matching under incongruity in conflict-related processing (Draschkow et al., 2018; Hamm et al., 2002; Truman & Mudrik, 2018). We note that a centro-parietal negativity in this latency range is variably labeled across paradigms (e.g., N2, N300, or N400); here, we use “N3” as a descriptive label and interpret it as an intermediate perceptual–semantic integration stage rather than early sensory encoding. In the present study, the N3 component showed significant differences across conditions, further supporting previous research on emotion processing and attention allocation mechanisms. Specifically, prosocial words elicited significantly larger N3 amplitudes than self-relevant and non-prosocial words, indicating that individuals invest more cognitive resources in socially meaningful stimuli during early semantic matching and perceptual semantic integration. This result is consistent with existing findings that the N3 reflects perceptual processing prior to semantic integration conflicts and is also involved in the biased processing of contextual information (Draschkow et al., 2018; McPherson & Holcomb, 1999; Mudrik et al., 2010; Hamm et al., 2002; Võ & Wolfe, 2013).

In addition, the congruence of task conditions significantly influenced N3 amplitudes: amplitudes were smaller in incongruent trials than in congruent trials. Importantly, this effect was qualified by a significant interaction between condition and electrode site, with congruent trials eliciting larger N3 amplitudes specifically at frontocentral electrodes (Fz and FCz). This suggests that when there is a conflict between stimulus and task goals, early semantic processing is inhibited or modulated, reflecting the interfering effect of cognitive conflict on attention allocation. This finding aligns with observations from Stroop task studies that the N3 is involved in initial object recognition and semantic matching/mismatching processing (Truman & Mudrik, 2018; X. Liu et al., 2021), indicating that task congruence may affect the allocation of attentional resources during early processing stages. Notably, N3 amplitudes elicited under the red songs condition were significantly larger than those under the neutral songs condition, demonstrating that the emotional state evoked by music can modulate the early processing of lexical stimulus in subsequent implicit association tasks. This result is consistent with previous research that emotional states influence attention allocation and conflict control processing (Y. Liu et al., 2020; Carretié et al., 1997; X. Liu et al., 2021). Specifically, relevant studies have shown that identification with red music can arouse self-transcendent emotions such as awe, which in turn influence prosocial tendencies through these social emotions (He et al., 2025). Thus, the larger N3 amplitudes under the red songs condition may be interpreted as reflecting differences in early processing of prosocial vocabulary, although arousal-driven modulation cannot be excluded. Nevertheless, because emotional arousal was not fully matched across musical stimuli, the N3 enhancement may reflect the combined influence of heightened arousal and culturally embedded meaning.

Furthermore, no significant interaction between music training experience and song type was observed for N3 amplitudes, and no main effect of music training was detected. This indicates that the regulatory effect of red songs on early semantic processing does not depend on an individual’s music training background. From the perspective of the time course of information processing, earlier ERP components typically reflect automatic, stimulus-driven processing, while individual difference factors are more likely to manifest in later stages (Polich, 2007; Kok, 2001). Therefore, this result suggests that the emotional arousal and social significance triggered by red songs may be associated with differences in early processing of prosocial information, possibly in a relatively automatic manner, rather than relying on high-order auditory analysis or cognitive strategies formed through long-term music training.

Finally, the LPC (500–1000 ms, parietal sites) showed a significant main effect of group, with the music training group exhibiting larger amplitudes. The LPC reflects high-level emotional encoding and motivational value processing (Coates & Campbell, 2010; Lou et al., 2021). In the present study, a significant main effect of song type was observed, with larger LPC amplitudes following red songs than neutral songs. In addition, a main effect of electrode site was found, with the largest amplitudes at parietal regions (Pz) and the smallest at frontocentral sites (FCz), indicating a typical posterior distribution of LPC activity. In contrast, no significant main effect of music training and no interaction between music training and song type were observed for LPC amplitudes. This pattern suggests that LPC modulation in the present study is primarily driven by stimulus-related factors (i.e., song type) rather than individual differences in musical expertise. Previous studies have attributed larger LPC amplitudes in musicians primarily to enhanced emotional and motivational processing (Herholz & Zatorre, 2012; Schellenberg et al., 2015), and the effect may also reflect greater cognitive resources or engagement with the structural and informational content of musical stimulus more generally. For instance, musicians show enhanced neural responses to critical notes in melodies, reflecting heightened sensitivity to musical structure rather than just emotional valence (Centanni et al., 2020). Similarly, expertise influences the decisional and recognition aspects of musical processing, with ERP amplitudes differing between musicians and nonmusicians based on familiarity and task demands (Besson & Faïta, 1995). Moreover, musicians generally exhibit more accurate and heightened perception of musical features such as rhythm, harmony, and intensity, which facilitates stronger emotional responses and neural activation in music processing (Bhatara et al., 2011; Blood & Zatorre, 2001; Juslin & Laukka, 2003). However, given the absence of music training effects in the present data, these interpretations remain speculative and are not directly supported by the current findings. Together, these ERP findings suggest a possible multi-stage neurocognitive framework, in which red songs are associated with differences in early perceptual attention (N1), attentional allocation to cognitive conflict (P2), and semantic–emotional processing of prosocial concepts (N3), as well as later-stage evaluative processing (LPC). Across these stages, the effects appear to be primarily driven by song type, with limited evidence for modulation by music training at the neural level. These complementary mechanisms may jointly contribute to the observed behavioral effects.

In conclusion, the present study demonstrates that the implicit prosocial effects of red songs are modulated by music training experience, with distinct neural mechanisms underlying different stages of cognitive–emotional processing (Notably, no significant “red song × music training” interaction was detected in the ERP data analyses, and no main effect of music training was observed across ERP components.), indicating that the behavioral finding concerning the moderating role of music training in the implicit prosocial effects of red songs receives only partial neurophysiological support. The findings highlight the complexity of cultural music’s social impacts, underscore the importance of individual differences in shaping media effects, and provide a foundation for future research on music, cultural context, and prosocial behavior. By bridging music psychology, cultural studies, and cognitive neuroscience, this research not only enriches theoretical frameworks but also offers practical strategies for leveraging culturally specific music to promote prosociality in educational and intervention settings.

### 4.3. Dissociation Between Implicit and Explicit Prosocial Tendencies

Pearson’s correlation analysis showed a lack of significant associations between explicit prosocial tendency scores and implicit measures (SC-IAT D-scores, all *p* > 0.05), suggesting a distinction between implicit and explicit prosocial constructs, which is consistent with the dual attitude model (Wilson et al., 2000; Aydinli et al., 2014). This dissociation underscores that red songs and music training modulate implicit prosociality through automatic, pre-conscious mechanisms (semantic priming, emotional resonance, attentional bias) rather than conscious deliberative processes (Wilson et al., 2000; Greenwald et al., 1998). This finding aligns with prior research showing that prosocial media (including music) primarily impacts implicit rather than explicit prosocial outcomes (Ding et al., 2015; Hong et al., 2023), highlighting the importance of measuring implicit tendencies to fully understand music’s social effects.

### 4.4. Limitations and Future Directions

While the present findings offer valuable insights into the interplay between red songs, music training, and implicit prosocial attitudes, three key limitations represent important avenues for future refinement, grounded in the scope and design of the current research:

First, the study focused exclusively on immediate post-exposure effects of red song listening, leaving the durability and generalizability of the observed effects unaddressed. The enhanced implicit prosocial D-scores and modulated ERP components (e.g., N1, N3) may reflect transient cognitive–emotional priming—driven by the salience of red songs’ cultural and emotional cues—rather than stable, long-term shifts in implicit attitudes. Without longitudinal follow-up, it remains unclear whether these effects persist beyond hours or days, or whether repeated exposure might strengthen, stabilize, or even attenuate them over time. Future research should adopt longitudinal designs to track dynamic changes in implicit prosocial indicators. Such designs would clarify whether sustained engagement with red songs consolidates prosocial attitudes, how the interaction between music training and red songs evolves with repeated exposure, and whether effects endure after exposure ceases—critical for validating the practical utility of red songs in prosocial intervention.

Second, the stimulus set and failure to disentangle lyrical and melodic contributions limit the interpretability of the findings. The study relied on a single red song (“China in the Lantern Light”) and one neutral song (“Lake Baikal”), raising the possibility that results are stimulus-specific rather than generalizable to red songs as a category. Red songs vary widely in thematic focus (e.g., patriotism vs. collective unity) and musical features (e.g., tempo, mode), and the selected red song may possess unique attributes (e.g., particularly vivid lyrical imagery or arousing melody) that drive effects. Additionally, while we matched songs on basic acoustic features, we did not systematically isolate the independent contributions of lyrics (semantic content advocating prosocial values), melody (emotional arousal from musical structure) and other musical dimensions (e.g., harmony, orchestration arrangement, and vocal timbre). These structural and acoustic differences may independently influence early auditory and attentional ERP components (e.g., N1 and P2). Importantly, the red song exhibited significantly higher emotional arousal than the neutral song in the material validation phase. While this arousal difference may have contributed to early ERP modulations (e.g., N1), the absence of a uniform main effect of song type in behavioral outcomes suggests that arousal alone may not sufficiently explain the observed interaction with music training. Therefore, it remains possible that the effects observed in the present study reflect the combined influence of emotional arousal and culturally embedded prosocial/revolutionary meaning, rather than purely semantic content alone. Future research should more strictly match stimuli on arousal dimensions or experimentally manipulate arousal levels within the same musical material to disentangle content-specific and arousal-driven mechanisms. The larger N1 and N3 amplitudes elicited by red songs, for example, could stem from lyrical priming, melodic resonance, or their interaction—an ambiguity that hinders mechanistic understanding. Future research should expand the stimulus set to include three to five red songs (diverse themes/styles) and three to five neutral songs (strictly matched on acoustic features), while incorporating “lyrics-only” (instrumental-free) and “melody-only” (lyric-free) conditions to disentangle these components. More comprehensive acoustic matching procedures and assessments of participants’ familiarity with song lyrics would further improve causal interpretability. Future work should employ multiple exemplars per category and acoustically matched stimuli (or controlled instrumental and lyric manipulations) to improve causal interpretability.

Third, sample homogeneity and insufficiently nuanced measurement of music training restrict external validity. The sample comprised only Chinese college students with a shared collectivist cultural background, and the musical stimuli (red songs) are themselves deeply embedded within China-specific revolutionary narratives and collective value systems. This raises questions about whether effects generalize to other populations, particularly those without similar historical, educational, or cultural exposure to such ideological musical forms. The prosocial modulation observed in the present study is therefore likely context-dependent and may rely on culturally internalized meanings associated with revolutionary spirit, patriotism, and collective identity. Age cohort differences may play an important role. Older generations, who may possess stronger autobiographical or historically grounded connections to red songs, could exhibit qualitatively different cognitive–emotional responses compared to contemporary college students. Conversely, individuals from non-Chinese cultural contexts may lack the sociohistorical framework necessary for similar associative activation. Regarding individual differences, our operationalization of music training—defined as ≥5 years of formal training—did not differentiate between training types (instrumental and vocal), intensity (e.g., 1 and 5 h/week), or quality (professional and amateur). These distinctions may modulate sensitivity to red songs’ cues: vocal training, for instance, might enhance lyrical processing, while instrumental training could strengthen melodic perception—potentially leading to distinct patterns of socio-cognitive engagement with prosocial themes. Because these dimensions were not separately assessed, within-group heterogeneity may have attenuated more specific effects rather than allowing precise identification of modality-specific influences. Informal music exposure (e.g., frequent listening, non-formal choir participation) was also unmeasured, which may have contributed additional variability within groups. Future research should recruit diverse samples across age, education, and cultural background, and extend cross-cultural comparisons to examine whether similar effects emerge in societies without comparable revolutionary musical traditions. Refining music training assessment by capturing type, intensity, and duration, while controlling for informal exposure to isolate the unique role of formal musical expertise. Addressing these limitations will strengthen the robustness, generalizability, and mechanistic depth of future research, further advancing our understanding of how cultural music shapes implicit prosocial cognition and informing evidence-based prosocial education strategies.

## 5. Conclusions

Using the SC-IAT paradigm and ERP technology, this study found no significant main effects of red songs or music training on implicit prosocial attitudes, but their interaction was statistically significant: individuals with music training exhibited stronger implicit prosocial attitudes under the red songs condition, whereas the non-music training group showed an opposite, non-significant trend. Neurally, red songs elicited larger N1, P2, and N3 amplitudes relative to neutral songs—patterns that may be consistent with differences in early perceptual attention (N1), attentional allocation to motivationally relevant stimulus (P2; potentially reflecting cognitive conflict in incongruent trials, which aligns with the prosocial-self association task design), and deeper semantic–emotional processing of prosocial concepts (N3). Notably, no significant “red song × music training” interactions were observed for ERP data. In contrast, music training was associated with larger LPC amplitudes, a pattern consistent with enhanced high-level emotional encoding. Collectively, these findings provide preliminary evidence for the conditional nature of red songs’ prosocial effects (moderated by music training) and provide preliminary evidence regarding the underlying neurocognitive mechanism, offering theoretical insights and preliminary practical guidance for prosocial education and cultural interventions.

## Figures and Tables

**Table 1:** Results of mixed-design ANOVA on D-scores.

Source of Variation	F(1, 58)	*p*-Value	η_p_^2^ (Partial Eta Squared)
Main effect of music training experience	0.41	0.52	0.01
Main effect of song type	0.67	0.42	0.01
Interaction effect (group × song type)	4.09	0.04	0.07

**Table 2:** Correlation analysis between explicit prosocial behaviors and implicit prosocial attitudes in the context of red song genres.

Variables	M ± SD	1	2	3	4
1. SC-IAT D-scores (red songs condition)	0.31 ± 0.35	1.00			
2. SC-IAT D-scores (neutral songs condition)	0.24 ± 0.48	0.14	1.00		
3. PTM scores (red songs condition)	97.15 ± 15.04	−0.14	0.39	1.00	
4. PTM scores (neutral songs condition)	94.27 ± 15.37	−0.09	0.06	0.89 ***	1.00

Note. SC-IAT, Single-Category Implicit Association; PTM, Prosocial Attitudes Measure. *** *p* < 0.001.

## Data Availability

The original contributions presented in this study are included in the article. Further inquiries can be directed to the corresponding author.

## References

[B1-behavsci-16-00505] Aydinli A., Bender M., Chasiotis A., Cemalcilar Z., van de Vijver F. J. R. (2014). When does self-reported prosocial motivation predict helping? The moderating role of implicit prosocial motivation. Motivation and Emotion.

[B2-behavsci-16-00505] Barry R. J., Clarke A. R., Johnstone S. J. (2003). A review of electrophysiology in attention-deficit/hyperactivity disorder: I. Qualitative and quantitative electroencephalography. Clinical Neurophysiology.

[B3-behavsci-16-00505] Begleiter H., Platz A. (1969). Cortical evoked potentials to semantic stimuli. Psychophysiology.

[B4-behavsci-16-00505] Bernat E., Bunce S., Shevrin H. (2001). Event-related brain potentials differ entiate positive and negative mood adjectives during both supraliminal and subliminal visual processing. International Journal of Psychophysiology.

[B5-behavsci-16-00505] Besson M., Faïta F. (1995). An event-related potential (ERP) study of musical expectancy: Comparison of musicians with nonmusicians. Journal of Experimental Psychology: Human Perception and Performance.

[B6-behavsci-16-00505] Bhatara A., Tirovolas A. K., Duan L. M., Levy B., Levitin D. J. (2011). Perception of emotional expression in musical performance. Journal of Experimental Psychology: Human Perception and Performance.

[B7-behavsci-16-00505] Blood A. J., Zatorre R. J. (2001). Intensely pleasurable responses to music correlate with activity in brain regions implicated in reward and emotion. Proceedings of the National Academy of Sciences.

[B8-behavsci-16-00505] Braboszcz C., Delorme A. (2011). Lost in thoughts: Neural markers of low alertness during mind wandering. Neuroimage.

[B9-behavsci-16-00505] Carlson R. W., Aknin L. B., Liotti M. (2016). When is giving an impulse? An ERP investigation of intuitive prosocial behavior. Social Cognitive and Affective Neuroscience.

[B10-behavsci-16-00505] Carretié L., Iglesias J., García T., Ballesteros M. (1997). N300, P300 and the emotional processing of visual stimuli. Electroencephalography and Clinical Neurophysiology.

[B11-behavsci-16-00505] Castro S. L., Lima C. F. (2014). Age and musical expertise influence emotion recognition in music. Music Perception.

[B12-behavsci-16-00505] Centanni T. M., Halpern A. R., Seisler A. R., Wenger M. J. (2020). Context-dependent neural responses to minor notes in frontal and temporal regions distinguish musicians from nonmusicians. Cognitive, Affective, & Behavioral Neuroscience.

[B13-behavsci-16-00505] Chen O. T., Chen J. (2022). Impact of belief in communism on moral cognition and its mechanism. Psychological Exploration.

[B14-behavsci-16-00505] Cheng Z. (2025). Research on the normalization and institutionalization of red song integration into college students’ ideal and belief education: A case study of “ideology, morality and the rule of law”. Voice of the Yellow River.

[B15-behavsci-16-00505] Coates M. A., Campbell K. B. (2010). Event-related potential measures of processing during an implicit association test. Neuroreport.

[B16-behavsci-16-00505] Crowley K. E., Colrain I. M. (2004). A review of the evidence for P2 being an independent component process: Age, sleep and modality. Clinical Neurophysiology.

[B17-behavsci-16-00505] Delorme A., Makeig S. (2004). EEGLAB: An open source toolbox for analysis of single-trial EEG dynamics including independent component analysis. Journal of Neuroscience Methods.

[B18-behavsci-16-00505] Ding F., Zhang L., Tan C. (2015). The influence of songs with pro-social lyrics on implicit and explicit pro-social behaviors of junior high school students. Journal of Linyi University.

[B19-behavsci-16-00505] Draschkow D., Heikel E., Võ M. L., Fiebach C. J., Sassenhagen J. (2018). No evidence from MVPA for different processes underlying the N300 and N400 incongruity effects in object-scene processing. Neuropsychologia.

[B20-behavsci-16-00505] Eisenberg N., Fabes R. A., Murphy B., Maszk P., Smith M., Karbon M. (1995). The role of emotionality and regulation in children’s social functioning: A longitudinal study. Child Development.

[B21-behavsci-16-00505] Evans K. M., Federmeier K. D. (2007). The memory that’s right and the memory that’s left: Event-related potentials reveal hemispheric asymmetries in the encoding and retention of verbal information. Neuropsychologia.

[B22-behavsci-16-00505] Faul F., Erdfelder E., Lang A.-G., Buchner A. (2007). G*Power 3: A flexible statistical power analysis program for the social, behavioral, and biomedical sciences. Behavior Research Methods.

[B23-behavsci-16-00505] Finnigan S., O’Connell R. G., Cummins T. D., Broughton M., Robertson I. H. (2011). ERP measures indicate both attention and working memory encoding decrements in aging. Psychophysiology.

[B24-behavsci-16-00505] Forbes C. E., Cameron K. A., Grafman J., Barbey A., Solomon J., Ritter W., Ruchkin D. S. (2012). Identifying temporal and causal contributions of neural processes underlying the implicit association test (IAT). Frontiers in Human Neuroscience.

[B25-behavsci-16-00505] Greenwald A. G., McGhee D. E., Schwartz J. L. (1998). Measuring individual differences in implicit cognition: The implicit association test. Journal of Personality and Social Psychology.

[B26-behavsci-16-00505] Greitemeyer T. (2009). Effects of songs with prosocial lyrics on prosocial behavior: Further evidence and a mediating mechanism. Personality & Social Psychology Bulletin.

[B27-behavsci-16-00505] Hahn A., Gawronski B., Wright J. D. (2015). Implicit social cognition. International encyclopedia of the social and behavioral sciences.

[B28-behavsci-16-00505] Hamm J. P., Johnson B. W., Kirk I. J. (2002). Comparison of the N300 and N400 ERPs to picture stimuli in congruent and incongruent contexts. Clinical Neurophysiology: Official Journal of the International Federation of Clinical Neurophysiology.

[B29-behavsci-16-00505] He Y., Wang S., Liu B., Wang P., Yang J., Zheng M. (2025). Red musical identity and subjective wellbeing: A longitudinal study of the chain mediating roles of awe and prosocial behavior. Frontiers in Psychology.

[B30-behavsci-16-00505] Herholz S. C., Zatorre R. J. (2012). Musical training as a framework for brain plasticity: Behavior, function, and structure. Neuron.

[B31-behavsci-16-00505] Hofmann W., Gawronski B., Gschwendner T., Le H., Schmitt M. (2005). A meta-analysis on the correlation between the implicit association test and explicit self-report measures. Personality & Social Psychology Bulletin.

[B32-behavsci-16-00505] Hong M., Liang D., Lu T. (2023). “Fill the world with love”: Songs with prosocial lyrics enhance online charitable donations among Chinese adults. Behavioral Sciences.

[B33-behavsci-16-00505] Huntjens R. J., Rijkeboer M. M., Krakau A., de Jong P. J. (2014). Implicit versus explicit measures of self-concept of self-control and their differential predictive power for spontaneous trait-relevant behaviors. Journal of Behavior Therapy and Experimental Psychiatry.

[B34-behavsci-16-00505] Jing Y., Xu Z., Pang Y., Liu X., Zhao J., Liu Y. (2024). The neural correlates of food preference among music kinds. Foods.

[B35-behavsci-16-00505] Juslin P., Laukka P. (2003). Communication of emotions in vocal expression and music performance: Different channels, same code?. Psychological Bulletin.

[B36-behavsci-16-00505] Kanske P., Kotz S. A. (2007). Concreteness in emotional words: ERP evidence from a hemifield study. Brain Research.

[B37-behavsci-16-00505] Kanske P., Plitschka J., Kotz S. A. (2011). Attentional orienting towards emotion: P2 and N400 ERP effects. Neuropsychologia.

[B38-behavsci-16-00505] Karpinski A., Steinman R. B. (2006). The single category implicit association test as a measure of implicit social cognition. Journal of Personality and Social Psychology.

[B39-behavsci-16-00505] Keltner D., Haidt J. (2003). Approaching awe, a moral, spiritual, and aesthetic emotion. Cognition & Emotion.

[B40-behavsci-16-00505] Key A. P. F., Dove G. O., Maguire M. J. (2010). Linking brainwaves to the brain: An ERP primer. Developmental Neuropsychology.

[B41-behavsci-16-00505] Kok A. (2001). On the Utility of P3 amplitude as a measure of processing capacity. Psychophysiology.

[B42-behavsci-16-00505] Konečni V. J. (2008). Does music induce emotion? A theoretical and methodological analysis. Psychology of Aesthetics, Creativity, and the Arts.

[B43-behavsci-16-00505] Kou Y., Hong H. F., Tan C., Li L. (2007). Revisioning the prosocial tendencies measure for adolescents. Psychological Development and Education.

[B44-behavsci-16-00505] Li W. F., Jiang C. Q., Li C. C., Liu Y., Liu F. B. (2015). Spatio-temporal characteristics of human brain processing of self-referential tasks: Evidence from ERPs. Psychological Exploration.

[B45-behavsci-16-00505] Liu X., Liu Y., Shi H., Li L., Zheng M. (2021). Regulation of Mindfulness-based music listening on negative emotions related to COVID-19: An ERP study. International Journal of Environmental Research and Public Health.

[B46-behavsci-16-00505] Liu Y., Zhang L., Jackson T., Wang J., Yang R., Chen H. (2020). Effects of negative mood state on event-related potentials of restrained eating subgroups during an inhibitory control task. Behavioural Brain Research.

[B47-behavsci-16-00505] Liu Y., Zhao J., Zhang X., Gao X., Xu W., Chen H. (2019). Overweight adults are more impulsive than normal weight adults: Evidence from ERPs during a chocolate-related delayed discounting task. Neuropsychologia.

[B48-behavsci-16-00505] Lou Y., Lei Y., Astikainen P., Peng W., Otieno S., Leppänen P. H. T. (2021). Brain responses of dysphoric and control participants during a self-esteem implicit association test. Psychophysiology.

[B49-behavsci-16-00505] Ma F., Sun Y. (2025). The study on how red culture shapes adolescents’ values. The Party Building and Ideological Education in Schools.

[B50-behavsci-16-00505] McPherson W. B., Holcomb P. J. (1999). An electrophysiological investigation of semantic priming with pictures of real objects. Psychophysiology.

[B51-behavsci-16-00505] Mudrik L., Lamy D., Deouell L. Y. (2010). ERP evidence for context congruity effects during simultaneous object-scene processing. Neuropsychologia.

[B52-behavsci-16-00505] Nosek B. A., Hawkins C. B., Frazier R. S. (2011). Implicit social cognition: From measures to mechanisms. Trends in Cognitive Sciences.

[B53-behavsci-16-00505] Pichon I., Boccato G., Saroglou V. (2007). Nonconscious influences of religion on prosociality: A priming study. European Journal of Social Psychology.

[B54-behavsci-16-00505] Piff P. K., Dietze P., Feinberg M., Stancato D. M., Keltner D. (2015). Awe, the small self, and prosocial behavior. Journal of Personality and Social Psychology.

[B55-behavsci-16-00505] Polich J. (2007). Updating P300: An integrative theory of P3a and P3b. Clinical Neurophysiology.

[B56-behavsci-16-00505] Proverbio A. M., Camporeale E., Brusa A. (2020). Multimodal recognition of emotions in music and facial expressions. Frontiers in Human Neuroscience.

[B57-behavsci-16-00505] Qin J. (2024). Research on the application of red songs in music education in colleges and universities under the concept of curriculum thinking and politics. World Journal of Educational Research.

[B58-behavsci-16-00505] Rodríguez-Gómez P., Martín-Loeches M., Colmenares F., Romero Ferreiro M. V., Moreno E. M. (2020). He had it Comin’: ERPs Reveal a Facilitation for the Processing of Misfortunes to Antisocial Characters. Cognitive, Affective & Behavioral Neuroscience.

[B59-behavsci-16-00505] Ruth N. (2019). “If you wanna make the world a better place”: Factors influencing the effect of songs with prosocial lyrics. Psychology of Music.

[B60-behavsci-16-00505] Schacht A., Sommer W. (2009). Time course and task dependence of emoti on effects in word processing. Cognitive, Affective & Behavioral Neuroscience.

[B61-behavsci-16-00505] Schellenberg E. G., Corrigall K. A., Dys S. P., Malti T. (2015). Group music training and children’s prosocial skills. PLoS ONE.

[B62-behavsci-16-00505] Schroeder D. A., Graziano W. G. (2015). The field of prosocial behavior: An introduction and overview. The Oxford handbook of prosocial behavior.

[B63-behavsci-16-00505] Stellar J. E., Gordon A. M., Piff P. K., Cordaro D., Anderson C. L., Bai Y., Maruskin L. A., Keltner D. (2017). Self-transcendent emotions and their social functions: Compassion, gratitude, and awe bind us to others through prosociality. Emotion Review.

[B64-behavsci-16-00505] Truman A., Mudrik L. (2018). Are incongruent objects harder to identify? The functional significance of the N300 component. Neuropsychologia.

[B65-behavsci-16-00505] Võ M. L., Wolfe J. M. (2013). Differential electrophysiological signatures of semantic and syntactic scene processing. Psychological Science.

[B66-behavsci-16-00505] Wen F. F., Zuo B. (2007). The measurement of implicit social cognition to assess the single attitudes object. Advances in Psychological Science.

[B67-behavsci-16-00505] Williams J. K., Themanson J. R. (2011). Neural correlates of the implicit association test: Evidence for semantic and emotional processing. Social Cognitive and Affective Neuroscience.

[B68-behavsci-16-00505] Wilson T. D., Lindsey S., Schooler T. Y. (2000). A model of dual attitudes. Psychological Review.

[B69-behavsci-16-00505] Xiao F., Zheng Z., Wang Y., Cui J., Chen Y. (2015). Conflict monitoring and stimulus categorization processes involved in the prosocial attitude implicit association test: Evidence from event-related potentials. Social Neuroscience.

[B70-behavsci-16-00505] Xu M., Yang Q., Shi Y. (2025). The educational value and practical approaches of red music culture in private colleges under the background of the new era. Modern Music.

[B71-behavsci-16-00505] Xu Q., Liu S., Li M., Wang X., Li J., Yuan X., Yang M., Yang M., Jiang Z., Gou Q., Liu N., Han J., Yang D., Ren X. (2025). The impact of songs with prosocial lyrics on implicit cognition and prosocial behavior: A prospective event-related brain potential study. Frontiers in Psychology.

[B72-behavsci-16-00505] Yaden D. B., Kaufman S. B., Hyde E., Chirico A., Gaggioli A., Zhang J. W., Keltner D. (2019). The development of the Awe Experience Scale (AWE-S): A multifactorial measure for a complex emotion. The Journal of Positive Psychology.

[B73-behavsci-16-00505] Zheng M. (2021). On the emotional experience of the centennial red songs of the communist party of China. Journal of Southwest University (Social Sciences).

[B74-behavsci-16-00505] Zhou L., Yang Y., Li S. (2022). Music-induced emotions influence intertemporal decision making. Cognition & Emotion.

